# Mental health of the Moroccan population in crisis situations: Prevalence of anxiety and depressive disorders and associated factors

**DOI:** 10.1192/j.eurpsy.2026.11325

**Published:** 2026-07-31

**Authors:** S. Essadki, F. Wahoud, R. Lamsyah, W. Hajjaji, A. Zarouali, O. El Mouhib, M. El Khalloufi, K. M’srouh, M. A. Lafraxo, S. Bechrouri

**Affiliations:** 1 Higher Institute of Nursing Professions and HealthTechniques; 2 Laboratory of Maternal, Infant, and Mental Health(LSMIM), Faculty of Medicine and Pharmacy, Oujda; 3 Higher Institute of Nursing Professions and HealthTechniques, Fez; 4 Laboratory of Biology and Health, Faculty of Science, Ibn Tofail University, Kenitra, Morocco

## Abstract

**Introduction:**

The world is facing recurrent episodes of crises—whether health-related, economic, natural, or geopolitical. Such situations can generate significant negative effects on the mental health of populations. Researchers have reported increased levels of anxiety and depression during these events.

**Objectives:**

This study aims to identify the crisis situations that have affected the mental health of Moroccans, whether they were directly or indirectly exposed to these crises.

**Methods:**

This is an analytical study conducted using an online questionnaire disseminated across various social media platforms. The instrument consists of two sections: the first includes sociodemographic characteristics of the participants as well as the crises they experienced; the second uses the Hospital Anxiety and Depression Scale (HADS). A total of 815 participants aged over 18 years completed the questionnaire.

**Results:**

Data analysis shows that 96.4% of participants presented clear anxious–depressive symptomatology. Sex, age, unemployment or financial insecurity, as well as direct or indirect exposure to the Al Haouz earthquake and the COVID-19 pandemic, were significantly associated with higher levels of anxiety and depression

**Conclusions:**

This study highlights the severity of the psychological impact of crisis situations on the Moroccan population and underscores the need for responsive and resilient mechanisms to prevent and provide psychological support to citizens.

**Disclosure of Interest:**

None Declared

